# Intermingled fates of the South China Sea and Philippine Sea plate

**DOI:** 10.1093/nsr/nwz107

**Published:** 2019-07-31

**Authors:** Minghui Zhao, Jean-Claude Sibuet, Jonny Wu

**Affiliations:** 1 Key Laboratory of Ocean and Marginal Sea Geology, South China Sea Institute of Oceanology, Chinese Academy of Sciences, China; 2 Innovation Academy of South China Sea Ecology and Environmental Engineering, Chinese Academy of Sciences, China; 3 Ifremer Centre de Brest, Plouzané Cedex, France; 4 Department of Earth and Atmospheric Sciences, University of Houston, USA

Recent studies have shown the extent and nature of the South China Sea (SCS) at the end of spreading by unfolding (i.e. structurally restoring) the Manila slab, which is the subducted part of the SCS, and by identifying the nature of the crust-lithosphere (oceanic or thinned continental) from mid-slab *P*-wave velocity perturbations (*dVp*) [1,2]. The objective of this paper is to propose a reconstruction of the SCS at the end of seafloor spreading and to discuss its geodynamic consequences in the context of the SCS and Philippine Sea plate (PSP) evolution. Reasonably accurate PSP paleo-latitudes and poorly defined paleo-declinations were primarily used to establish the kinematic evolution of the PSP through time (e.g. [3,4]) until 2016, when Wu *et al.* [1] introduced new kinematic constraints based on the unfolding and restoration of Southeast Asian slabs. Here, we propose to better constrain the relationship between the SCS, the Huatung basin (HB) and the PSP. Our main target is to bring new light on the challenging problem of SCS subduction initiation along a major shear-plate boundary. For that, we build on the new kinematic constraints provided by Wu *et al.* [1] and consider that the HB was not formed during Tertiary (e.g. [5,6]), but during the early Cretaceous (e.g. [7]).

## GEOMETRY OF THE SCS OCEANIC DOMAIN AT THE END OF SEAFLOOR SPREADING

The Manila subduction zone along the eastern boundary of the present-day SCS has undoubtedly played an important role in the evolutionary history of the SCS; however, the details are unclear because the eastern part of the SCS has already subducted. What did the SCS look like at the end of seafloor spreading (i.e. between 15.5 and 20 Ma) [8,9]? What were the size and shape of the SCS oceanic domain? What was the plate configuration at the inception of the Manila subduction zone?

**Figure 1.**
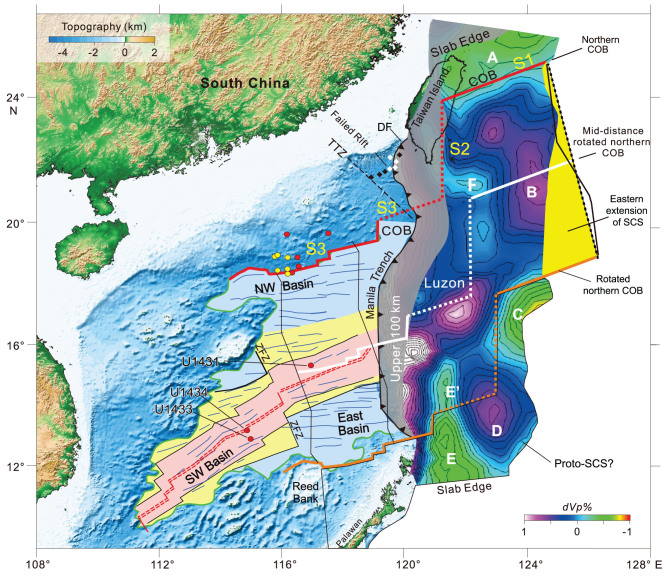
South China Sea (SCS) oceanic domain with unfolded Manila slab. The SCS oceanic domain, characterized by N055°, N075° and N085° seafloor-spreading directions, is delimited by pink, yellow and light-blue areas [11], respectively. The extinct spreading ridge (ESR, double red dashed line) in the East basin has been modified from Zhao *et al.* [12] and Zhong *et al.* [13]. The green line is the boundary of the oceanic domain from Sibuet *et al.* [11]. The northern continent–ocean boundary (COB) appears as a red line defined in Liu *et al.* [2], slightly modified for the segment crossing the Manila trench. Red and yellow circles are drilling sites from IODP Legs 349 and 367/368, respectively. The unfolded Manila slab is colored by intra-slab tomographic velocities *dVp* [1] and is attached to the SCS along the Manila trench. It extends 400–500 km east of the Manila trench. The N–S gray shadow mask (<100-km width) corresponds to probable artifacts and was not interpreted. Seafloor-spreading flow-lines are shown in black. The southern COB acquired in this study by rotating the northern COB with respect to Eurasia (EU) is shown in orange, and is consistent with that previously identified (in green). The mid-distance rotated northern COB (in white) follows the ESR. S1, S2 and S3 are COB segments. Areas A, B, C, D, E, E’ and F are discussed in the text. The yellow area is the extension of the Manila slab oceanic domain discussed in the text.

**Figure 2:**
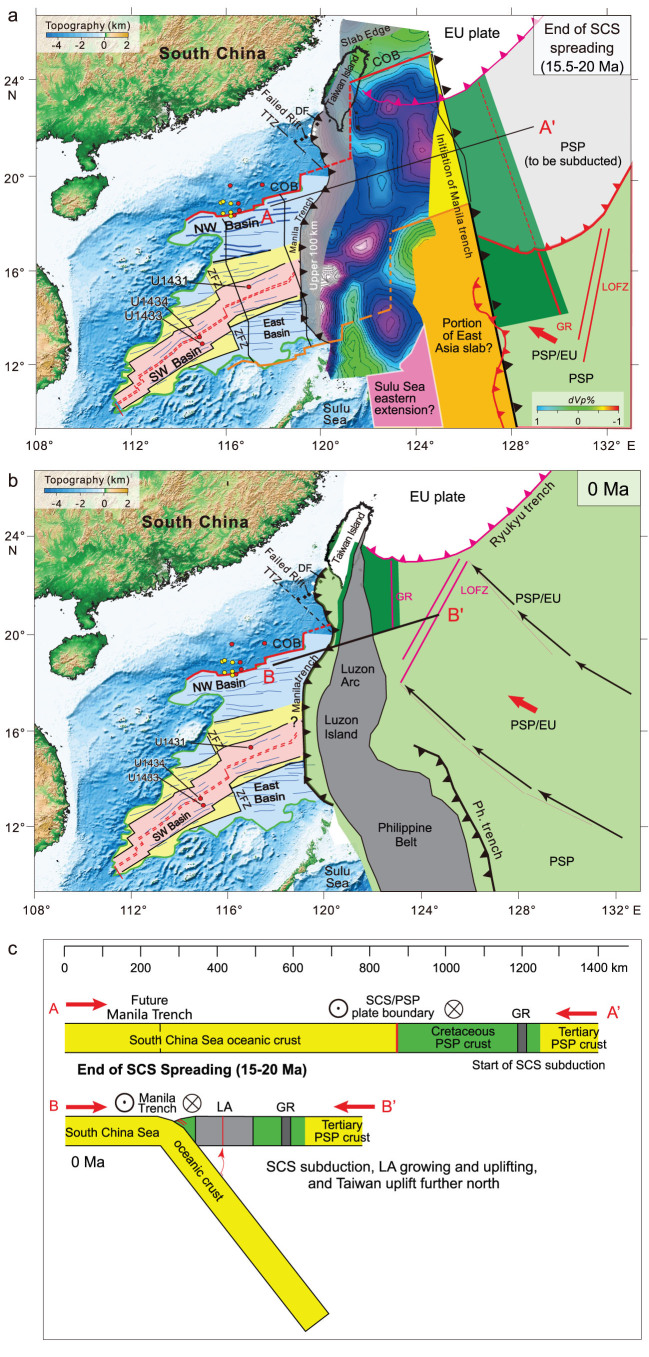
Kinematic evolution of SCS and PSP. Dark green, early Cretaceous HB; medium green, extension of the HB within the portion of PSP in light gray, which will subduct beneath EU; light green, present-day PSP; yellow domain, eastern extension of the SCS at the end of seafloor spreading; orange domain, portion of East Asian slabs defined in Wu *et al.* [1]; light-pink domain, northeastward extension of Sulu Sea. (a) End of SCS spreading (15.5–20 Ma): the inception of subduction along the former left-lateral shear-plate boundary of the young SCS oceanic crust beneath the PSP might have occurred before the end of SCS spreading. (b) Present-day situation with the emplacement of the Philippine belt and the accretion of the western part of the HB located west of the Luzon arc. (c) Simplified cross-sections AA’ and BB’ located in (a) and (b) (black lines).

Figure 1 shows the SCS domain when the Manila slab is unfolded and restored along the Manila trench. This solution is based on an MITP08 seismic tomography model augmented by the regional Chinese seismic network [1]. The continent–ocean boundary (COB) of the northern SCS consists of three segments (S1, S2 and S3). Based on *dVp* values within the mapped slab, slightly negative areas (in green) roughly correspond to thinned continental crust and positive areas (in blue) to oceanic crust. Thus, S1 corresponds to the northeastern portion of the northern COB within the slab, S3 is the portion of the northern COB defined by Liu *et al.* [2] and slightly modified for the part crossing the Manila trench and segment S2 joins S1 to S3 (dashed red line in Fig. 1). S2 is mostly located within the unreliable uppermost 100 km [1] of the Manila slab (gray stripe in Fig. 2). It is ~350 km long and its trend can be changed by +/−20° in azimuth, being approximately parallel to the Luzon arc when it collided with the Eurasian margin. Indeed, a ‘simultaneous’ Taiwan collision between 5 and 6 Ma was recently suggested by Taiwan zircon fission track data [10].

Based on spreading flow-lines (black lines in Fig. 1), the whole northern COB, rotated with respect to Eurasia (EU) by using Briais *et al.*’s [8] rotation parameters, corresponds to the COB previously identified as a green line on the southern margin of the SCS. Areas E’ and F located between the two COBs are not consistently reproduced in other tomographic models and thus are not reliable features [2]. We consequently suggest that the domain located between the two COBs is fully oceanic. The rotated northern COB located at the mid-distance between the two COBs closely matches the extinct spreading ridge (ESR; red double dashed lines in Fig. 1) even if several ridge jumps [8,9] have been identified. Note that the ESR track defined by Sibuet *et al.* [11] has been slightly modified from Zhao *et al.* [12] and in its eastern part, close to the Manila trench, where a seamount previously thought to be on the ESR is in fact 32 Ma old [13]. The good fit between the ESR and the mid-distance rotated COB (in white) increases the credibility of our proposed reconstruction of the entire SCS oceanic domain.

In addition, as the eastern boundary of the slab is not necessarily a plate tectonic boundary, the real eastward extension of the oceanic domain might follow an opening flow-line, acting as a left-lateral shear-plate boundary during the SCS opening. The resulting yellow domain would be consequently part of the SCS oceanic domain. Therefore, for the first time, we have established the real extent of the oceanic domain at the end of SCS spreading. This oceanic domain is bounded by the northern COB (S1, S2 and S3 segments, the green line, which circles the SW basin, the rotated northern COB and the easternmost flow-line located east of the yellow domain) (Fig. 1).

## THE INTERMINGLED FATES OF THE SCS AND PSP

The first episode of rifting along the northeastern SCS started in the earliest Eocene (56.0–40.0 Ma) with NNE–SSW trending (30°–45°) small rift basins [14,15]. The second episode of rifting began in the late Eocene (40.0–33.0 Ma) with a new set of 45°–70° trending rifted basins [14,15]. In the PSP, two major spreading phases are identified from magnetic lineations [16]. The first one began at Chron 24 (53.2 Ma), possibly earlier at Chron 25 (56.9 Ma) or 26 (58.6 Ma). However, these last two chrons are very short lineations and their potential conjugate chrons are absent in the northern PSP because the initial magnetic fabric was overprinted during the formation of the Oki–Daito ridge. The end of the first phase took place at Chron 20 (42.5 Ma). The second phase started at Chron 19 (40.4 Ma) and ended at Chron 13 (33.7 Ma) [16].

The first episode of the SCS rifting (56.0–40.0 Ma) corresponds to the first phase of the PSP seafloor spreading (~53.2–42.5 Ma). The second episode of the SCS rifting (40.0–33.0 Ma) corresponds to the second phase of the PSP spreading (~40.4–33.7 Ma). Therefore, during Eocene, similar-age tensional tectonic phases are observed on the SCS continental margin and in the oceanic PSP basin. We propose a simple sketch for the 56- to 33-Ma period, in which the two tensional domains of the SCS continental margins and of the PSP oceanic basin are connected by a left-lateral shear fault. We suggest that this fault is a large shear-plate boundary, which was active from 56 Ma up to now [1] and located between the PSP and the EU plate.

If paleo-latitude variations give the correct magnitude of the PSP northward component of the motion through time, the variations of paleo-magnetic declinations (synthesized in [1]) give the PSP eastward component of the motion but are unreliable because paleo-declination errors are up to 80° (e.g. [1,3]). It is still unclear whether the declinations show rotation of the entire PSP or local block rotations along the plate boundary. Therefore, even if unfolded slabs have low spatial and age resolutions relative to traditional plate tectonic constraints, large subducted slabs mapped around SE Asia supply important first-order insights (e.g. [1]). These slab constraints are independent from classical plate tectonic inputs and provide alternative insights that are of potential importance, given the lack of resolution on PSP paleo-declinations.

Since 56 Ma, a straightforward link between the SCS and PSP is not easily established given their 2200-km separation and the smaller size of the PSP relative to the present. Our interpreted large shear-plate boundary does not necessarily follow the same geological features through time. For example, magnetic lineations that show different trends across either side of the Gagua ridge indicate this boundary was not only a fracture zone (e.g. [3]), but a former plate boundary characterized by compressive features linked to slight changes in the PSP/EU poles of rotations [6]. Eakin *et al.* [17] and Deschamps *et al.* [18] confirm this view and suggest a limited westward underthrusting of the PSP beneath the HB probably occurring during early Oligocene at around 30 Ma, simultaneously with the cessation of the arc volcanism in the northern Philippine Islands [19]. Though the period of shear and failed subduction is not well defined along the Gagua ridge, it seems reasonable zone and failed subduction first followed the Gagua ridge [20] and then jumped to the west of Gagua ridge, i.e. to the east of the SCS as shown in Figs 1 and 2a.

An early Cretaceous age was proposed for the HB based on radiometric dating of gabbros belonging to the 115- to 125-Ma oceanic crust uplifted by the Miocene Luzon arc and the 115-Ma age of radiolarians deposited on this oceanic crust [7], suggesting the HB was aggregated to the younger PSP. The HB, including the Gagua ridge, is extrapolated to the position of the Ryukyu trench at the end of SCS spreading (Fig. 2a and c). The orange domain is probably a remaining fragment of the East Asian Sea oceanic slabs [1] and we suggest the pink area could correspond to the northeastern prolongation of the Sulu Sea. Therefore, it is tempting to propose the Manila trench initiated when SCS spreading stopped along the former left-lateral shear-plate boundary located where the lithospheric feature (i.e. thick black line striking NNW–SSE in Fig. 2a), active since the beginning of SCS spreading, separated the young SCS oceanic crust from the HB (Fig. 2a). However, remnants of the Miocene Luzon arc in the Coastal Range, Lanyu (an island of the Luzon arc) and northern Luzon suggest that this arc might have outcropped sinceearly Miocene as a result of the SCS subduction. If this is true, the SCS and its active spreading center might have partly subducted before the end of SCS opening.

This paper only considers a fairly narrow class of allowable solutions amongst the many plate models. For example, our proposed reconstruction model of Fig. 2a fits the gap left between the present-day SCS and the PSP in the 15-Ma reconstructions of Queaño *et al.* [4] and Hall [3]. The left-lateral shear-plate boundary became the incipient Manila subduction zone (Fig. 2a), with the younger SCS oceanic domain subducting below the older HB. Although tectonically counterintuitive, such a PSP/EU subduction configuration continues until today (Fig. 2b) with the formation of the Manila slab; the disappearance first of the fragment of the East Asian Sea oceanic slabs and then of the northeastern prolongation of the Sulu Sea; the formation of the Luzon arc; followed by collision of the northern Luzon arc with the N–S portion of the EU margin (segment S2) to produce Taiwan since ~6 Ma ago [10].

## THE SIGNIFICANCE OF DRILLING IN HUATUNG BASIN

Despite ample evidence the HB was formed during the early Cretaceous [1,7,20,21], many Earth scientists support HB formation during Tertiary, even as young as 15–30 Ma ago [22]. Implementation of a Tertiary or early Cretaceous-aged HB will produce wholly different East Asia kinematic reconstructions. The only way to validate kinematic reconstructions is to know the age and nature of the HB. A deeptowed magnetometer operated close to the sea bottom may be a solution. An older N–S deep-tow profile acquired by the Japanese at 1500-m depths in the northern HB was never fully published but did not show significantly better resolution than surface-ship magnetic measurements. Drilling in the HB would provide opportunities to sample and date the lower section of sediments below the terrigenous sediments eroded from Taiwan after its uplift (~6 Ma, [10]) but also the oceanic basement in order to characterize the nature and age of the oceanic crust. The fast >4-km/s stacked velocities of the lower section of sediments (e.g. [17]) suggest that these sediments are pelagic sediments deposited during the long northward journey of the HB since the early Cretaceous. These calcareous pelagic sediments might be similar to those found in Lanyu, an island of the Luzon arc [7].
